# Supplementary material for: Energy Homeostasis Gene Nucleotide Variants and Survival of Hemodialysis Patients—A Genetic Cohort Study
Source: J Clin Med. 2022 Sep 18;11(18):5477. doi: 10.3390/jcm11185477 (PMC9501434; doi:10.3390/jcm11185477)
Supplement: Supplementary file 1 [file jcm-11-05477-s001.zip › jcm-1815550-supplementary.pdf]

Supplementary Material

Energy homeostasis gene nucleotide variants and survival of hemodialysis patients – a genetic cohort study

Monika Katarzyna Świderska [1,2,*], Adrianna Mostowska [3], Damian Skrypnik [4], Paweł Piotr Jagodziński [3], Paweł Bogdański [4] and Alicja Ewa Grzegorzewska [3,†]

[1] Department of Nephrology, Transplantology and Internal Diseases, Poznan University of Medical Sciences, Przybyszewskiego 49, 60-355 Poznań, Poland
[2] Department of Diagnostic and Interventional Radiology and Nuclear Medicine, University Medical Center Hamburg-Eppendorf, Martinistraße 52, 20246 Hamburg, Germany
[3] Department of Biochemistry and Molecular Biology, Poznan University of Medical Sciences, Święcickiego 6, 60-781 Poznań, Poland
[4] Department of Treatment of Obesity, Metabolic Disorders, and Clinical Dietetics, Poznań University of Medical Sciences, Szamarzewskiego 82/84, 60-569 Poznań, Poland
* Correspondence: m.swiderska@uke.de; Tel.: +49-1522/281-5183
† The author died prior to the submission of this paper. This is one of her last works.

Table S1: Primer sequences and HRM conditions for the identification of genotyped nucleotide variants

Gene	rs no.	Alleles[1]	Primers for PCR[2] Amplification (5′–3′)	PCR product length (bp[3])	Annealing Temp. (°C)	Melt Temp. Range (°C)
FOXO3	rs2802292	G/T	F: AGGGTCTCTGTTGCTCACAAG<br>R: AGCAGGACCCCTTCATCTG	70	58	78 – 92
FOXO3	rs4946936	C/T	F: GAGTGCTTCCAGCATTAGGG<br>R: GGAGGGTTGCCTTTCTCAC	135	61	80 – 95
IGFBP3	rs3110697	A/G	F: ACGAGCAATGGCAATGGA<br>R: ACAGAATTGGCCTTTGGAGA	120	58	75 – 90
FABP1	rs2241883	C/T	F: GTGATTATGTCGCCGTTGAG<br>R: TGCAGACAGTGGTTCAGTTG	94	58	76 - 91
FABP1	rs2919872	C/T	F: GTGCACGTCCCAGTTCCT<br>R: AAGGTTGACGCCAAAGTCC	98	58	80 - 95
PCSK9	rs562556	A/G	F: TCAGCACACTCGGGGCCTAC<br>R: GCTCAGCAGCTCCTCATCTGG	142	58	80 – 95
PCSK9	rs11206510	C/T	F: GCTTCCCCAAGGATATAGGG<br>R: GGAAGAGGAGCCAAAGACG	68	58	75 – 90
ANGPTL6	rs8112063	C/T	F: GATCCAACCCATCTCAGCAG<br>R: AGTGGGGAGTAGCGGAGAC	96	58	80 – 95
DOCK6	rs737337	C/T	F: GGGTGCACAGAGGACACG<br>R: TGGGTGGACGGTCACAAG	62	58	80 – 95
DOCK6	rs17699089	A/G	F: TCATTCACGTTGTCGTCTCT<br>R: GTGGTTGCCACGTGTGTG	73	58	75 - 90

[1] underline denotes the minor allele
[2] PCR, polymerase chain reaction
[3] bp, base pairs

Table S2: The expected power for 1.00 – 1.75 ORs in association analyses of the HD patients who died and those who survived

				The expected power		
		Allele	Genotype	Genetic model		
Gene symbol	rs number	frequency[a]	relative risk	Additive	Dominant	Recessive
FOXO3	rs2802292	0.433	1.00	0.050	0.050	0.050
			1.25	1.000	0.896	0.782
			1.50	1.000	1.000	1.000
			1.75	1.000	1.000	1.000
FOXO3	rs4946936	0.345	1.00	0.050	0.050	0.050
			1.25	1.000	0.958	0.570
			1.50	1.000	1.000	0.988
			1.75	1.000	1.000	1.000
IGFBP3	rs3110697	0.429	1.00	0.050	0.050	0.050
			1.25	1.000	0.900	0.775
			1.50	1.000	1.000	1.000
			1.75	1.000	1.000	1.000
IGFBP3	rs2854744	0.457	1.00	0.050	0.050	0.050
			1.25	1.000	0.867	0.824
			1.50	1.000	1.000	1.000
			1.75	1.000	1.000	1.000
FABP1	rs2241883	0.348	1.00	0.050	0.050	0.050
			1.25	1.000	0.957	0.578
			1.50	1.000	1.000	0.989
			1.75	1.000	1.000	1.000
FABP1	rs2919872	0.466	1.00	0.050	0.050	0.050
			1.25	1.000	0.855	0.837
			1.50	1.000	1.000	1.000
			1.75	1.000	1.000	1.000
PCSK9	rs562556	0.179	1.00	0.050	0.050	0.050
			1.25	0.999	0.982	0.149
			1.50	1.000	1.000	0.448
			1.75	1.000	1.000	0.900
PCSK9	rs11206510	0.172	1.00	0.050	0.050	0.050
			1.25	0.998	0.981	0.139
			1.50	1.000	1.000	0.410
			1.75	1.000	1.000	0.833

ANGPTL6	rs8112063	0.434	1.00	0.050	0.050	0.050
			1.25	1.000	0.895	0.784
			1.50	1.000	1.000	1.000
			1.75	1.000	1.000	1.000
DOCK6	rs737337	0.073	1.00	0.050	0.050	0.050
			1.25	0.950	0.914	0.057
			1.50	1.000	1.000	0.080
			1.75	1.000	1.000	0.118
DOCK6	rs17699089	0.089	1.00	0.050	0.050	0.050
			1.25	0.974	0.943	0.063
			1.50	1.000	1.000	0.104
			1.75	1.000	1.000	0.174

[a] 1000 Genomes project, EUR samples.

Table S3: Frequency distributions of the analyzed nucleotide variants in hemodialysis patients and healthy controls

Gene symbol	rs number	HD patients				Healthy controls				P value[2]	
		Major homozygotes	Heterozygotes	Minor homozygotes	HWE[1], X^2 p value	Major homozygotes	Heterozygotes	Minor homozygotes	HWE[1], X^2 p value		
										0.253	Dominant
FOXO3	rs2802292	271 (34.6%)	396 (50.5%)	117 (14.9%)	0.155	137 (38.1%)	166 (46.1%)	57 (15.8%)	0.571	0.691	Recessive
										0.848	Additive
										0.782	Dominant
FOXO3	rs4946936	380 (48.8%)	324 (41.6%)	74 (9.5%)	0.682	179 (49.7%)	153 (42.5%)	28 (7.8%)	0.550	0.341	Recessive
										0.360	Additive
										0.728	Dominant
IGFBP3	rs3110697	266 (34.1%)	377 (48.3%)	137 (17.6%)	0.864	119 (33.1%)	185 (51.4%)	56 (15.6%)	0.253	0.401	Recessive
										0.640	Additive
										0.973	Dominant
IGFBP3	rs2854744	278 (35.5%)	376 (48.1%)	128 16.4%)	0.964	128 (35.7%)	174 (48.5%)	57 (15.9%)	0.867	0.835	Recessive
										0.861	Additive
										0.478	Dominant
FABP1	rs2241883	272 (34.8%)	377 (48.2%)	133 (17.0%)	0.903	133 (36.9%)	159 (44.2%)	68 (18.9%)	0.099	0.438	Recessive
										0.807	Additive
										0.368	Dominant
FABP1	rs2919872	234 (30.2%)	387 (49.9%)	154 (19.9%)	0.792	99 (27.6%)	184 (51.3%)	76 (21.2%)	0.579	0.612	Recessive
										0.404	Additive
										0.392	Dominant
PCSK9	rs562556	524 (67.2%)	240 (30.8%)	16 (2.1%)	0.055	251 (69.7%)	96 (26.7%)	13 (3.6%)	0.318	0.120	Recessive
										0.161	Additive
										0.818	Dominant
PCSK9	rs11206510	548 (70.2%)	210 (26.9%)	23 (2.9%)	0.597	255 (70.8%)	89 (24.7%)	16 (4.4%)	0.028	0.195	Recessive
										0.226	Additive
										0.219	Dominant
ANGPTL6	rs8112063	238 (30.5%)	370 (47.4%)	172 (22.1%)	0.215	97 (26.9%)	192 (53.3%)	71 (19.7%)	0.170	0.372	Recessive
										0.945	Additive

										0.198	Dominant
DOCK6	rs737337	625 (80.1%)	141 (18.1%)	14 (1.8%)	0.072	300 (83.3%)	57 (15.8%)	3 (0.8%)	0.872	0.213	Recessive
										0.195	Additive
										0.088	Dominant
DOCK6	rs176990893	553 (70.6%)	199 (25.4%)	31 (4.0%)	0.017	271 (75.5%)	80 (22.3%)	8 (2.2%)	0.471	0.134	Recessive
										0.106	Additive

[1] Hardy-Weinberg equilibrium

[2] Pearson's chi-squared test comparing frequency distributions of the analyzed variants in HD patients and healthy controls

Table S4: *DOCK6* rs737337 nucleotide variants and clinical and laboratory data of HD patients (n = 780)

Parameter	CC<br>n = 14	CT<br>n = 141	TT<br>n = 625	P value[1]	
Clinical data					
Male gender, n (%)	8 (57%)	78 (55%)	354 (57%)	0.795	Dominant
				0.956	Recessive
				0.970	Additive
Age at the beginning of RRT, years	61.8 (33.6 – 75.7)	62.2 (11.8 – 89.4)	61.2 (8.7 – 91.7)	0.649	Dominant
				0.906	Recessive
				0.784	Additive
RRT vintage, years	4.53 (1.39 – 23.64)	5.86 (0.09 – 28.67)	5.83 (0.04 – 32.88)	0.408	Dominant
				0.165	Recessive
				0.420	Additive
Renal transplantation, n (%)	3 (21%)	23 (16%)	123 (20%)	0.410	Dominant
				0.738[2]	Recessive
				0.745[2]	Additive
Diabetic nephropathy, n (%)	5 (36%)	57 (40%)	177 (28%)	0.005	Dominant
				0.771[2]	Recessive
				0.555[2]	Additive
Hypertensive nephropathy, n (%)	2 (14%)	21 (15%)	143 (23%)	0.029	Dominant
				0.745[2]	Recessive
				0.747[2]	Additive
Chronic glomerulonephritis, n (%)	1 (7%)	15 (11%)	85 (14%)	0.276	Dominant
				1.000[2]	Recessive
				0.706[2]	Additive
Chronic tubulointerstitial nephritis, n (%)	1 (7%)	11 (8%)	51 (8%)	0.864	Dominant
				1.000[2]	Recessive
				1.000[2]	Additive
Coronary artery disease, n (%)	7 (50%)	56 (40%)	231 (37%)	0.319	Dominant
				0.343	Recessive
				0.318	Additive
Myocardial infarction, n (%)	3 (21%)	30 (21%)	133 (21%)	0.998	Dominant
				1.000[2]	Recessive
				1.000[2]	Additive
Cerebral stroke, n (%)	4 (28%)	38 (27%)	163 (26%)	0.944	Dominant
				0.538[2]	Recessive
				0.537[2]	Additive
Dyslipidemia by K/DOQI, n (%)	8 (57%)	76 (54%)	325 (52%)	0.624	Dominant
				0.722	Recessive
				0.703	Additive
Hypolipemic therapy, n (%)	2 (14%)	60 (43%)	257 (41%)	0.534	Dominant
				0.236	Recessive
				0.229	Additive
Smoker, n (%)	0 (0%)	23 (16%)	109 (17%)	0.088	Dominant
				0.475[2]	Recessive
				0.143[2]	Additive
Weight, kg	70.4 (52.5 - 109)	71.5 (31.0 – 115.1)	73.2 (36.0 – 196.0)	0.108	Dominant
				0.944	Recessive
				0.114	Additive
Height, m	1.67 (1.50 – 1.80)	1.66 (1.28 – 1.91)	1.68 (1.37 – 1.93)	0.177	Dominant
				0.991	Recessive
				0.157	Additive
BMI, kg/m^2	25.7 (20.3 – 35.6)	25.1 (15.2 – 39.5)	25.8 (14.3 – 59.2)	0.124	Dominant
				0.776	Recessive
				0.216	Additive

Laboratory data					
CRP, mg/L	5.11 (1.30 – 52.5)	5.30 (0.29 – 96.60)	5.70 (0.10 – 195.00)	0.682<br>0.851<br>0.837	Dominant<br>Recessive<br>Additive
Total cholesterol, mg/dL	164.0 (109.3 – 280.0)	177.0 (72.0 – 363.0)	171.0 (76.3 – 626.0)	0.320<br>0.639<br>0.170	Dominant<br>Recessive<br>Additive
HDL-cholesterol, mg/dL	47.5 (27.0 – 68.0)	39.0 (17.3 – 81.0)	40.0 (6.0 – 118.0)	0.902<br>0.135<br>0.575	Dominant<br>Recessive<br>Additive
LDL-cholesterol, mg/dL	102.4 (38.5 – 211.0)	96.0 (31.0 – 328.0)	95.6 (25.0 – 512.0)	0.730<br>0.770<br>0.340	Dominant<br>Recessive<br>Additive
TG, mg/dL	141.0 (53.0 – 330.0)	149.0 (35.0 – 689.0)	149.0 (26.0 – 856.0)	0.448<br>0.956<br>0.240	Dominant<br>Recessive<br>Additive
Ca, mg/dL	8.51 (7.96 – 9.29)	8.80 (6.01 – 10.93)	8.84 (6.65 – 12.76)	0.199<br>0.104<br>0.187	Dominant<br>Recessive<br>Additive
Phosphate, mg/dL	4.91 (4.27 – 8.28)	5.04 (1.75 – 11.14)	5.15 (2.03 – 11.27)	0.291<br>0.952<br>0.436	Dominant<br>Recessive<br>Additive
Intact PTH, pg/mL	325.3 (88.0 – 796.2)	340.3 (12.9 – 3757.0)	402.0 (5.5 – 3118.2)	0.051<br>0.241<br>0.105	Dominant<br>Recessive<br>Additive
Calcium phosphate product, mg^2/dL^2	42.5 (34.8 – 69.8)	44.4 (16.7 – 87.4)	45.1 (18.4 – 108.7)	0.220<br>0.764<br>0.273	Dominant<br>Recessive<br>Additive

[1] Chi-squared test for dichotomous variables or Mann-Whitney U test for quantitative variables
[2] Fisher's exact test
Abbreviations: BMI: body mass index; CRP: C-reactive protein; HDL: high-density lipoprotein; K/DOQI: Kidney Disease Outcomes Quality Initiative; LDL: low-density lipoprotein, PTH: parathormone, RRT: renal replacement therapy; TG: triglycerides.

Table S5: *ANGPTL6* rs8112063 nucleotide variants and clinical and laboratory data of HD patients (n = 780)

Parameter	CC<br>N = 172	CT<br>N = 370	TT<br>N = 238	P value[1]	
Clinical data					
Male gender, n (%)	99 (58%)	211 (57%)	130 (55%)	0.504<br>0.730<br>0.555	Dominant<br>Recessive<br>Additive
Age at the beginning of RRT, years	62.1 (8.7 – 89.4)	61.0 (11.0 – 89.0)	61.2 (16.4 – 91.7)	0.433<br>0.824<br>0.784	Dominant<br>Recessive<br>Additive
RRT vintage, years	5.49 (0.04 – 29.88)	5.85 (0.09 – 31.09)	5.96 (0.15 – 32.88)	0.576<br>0.339<br>0.259	Dominant<br>Recessive<br>Additive
Renal transplantation, n (%)	36 (21%)	72 (19%)	41 (17%)	0.377<br>0.490<br>0.343	Dominant<br>Recessive<br>Additive
Diabetic nephropathy, n (%)	65 (38%)	110 (30%)	65 (27%)	0.166<br>0.024<br>0.024	Dominant<br>Recessive<br>Additive
Hypertensive nephropathy, n (%)	30 (17%)	78 (21%)	59 (25%)	0.127<br>0.151<br>0.075	Dominant<br>Recessive<br>Additive
Chronic glomerulonephritis, n (%)	18 (10%)	48 (13%)	35 (15%)	0.333<br>0.271<br>0.207	Dominant<br>Recessive<br>Additive
Chronic tubulointerstitial nephritis, n (%)	6 (3%)	37 (10%)	20 (8%)	0.825<br>0.012<br>0.044	Dominant<br>Recessive<br>Additive
Coronary artery disease, n (%)	63 (37%)	142 (38%)	88 (37%)	0.784<br>0.744<br>0.943	Dominant<br>Recessive<br>Additive
Myocardial infarction, n (%)	32 (19%)	83 (22%)	50 (21%)	0.947<br>0.354<br>0.548	Dominant<br>Recessive<br>Additive
Cerebral stroke, n (%)	42 (24%)	106 (29%)	56 (24%)	0.269<br>0.558<br>0.835	Dominant<br>Recessive<br>Additive
Dyslipidemia by K/DOQI, n (%)	82 (48%)	189 (51%)	137 (58%)	0.052<br>0.168<br>0.048	Dominant<br>Recessive<br>Additive
Hypolipemic therapy, n (%)	77 (45%)	153 (41%)	95 (40%)	0.511<br>0.350<br>0.326	Dominant<br>Recessive<br>Additive
Smoker, n (%)	30 (17)	62 (17%)	40 (17%)	0.837<br>0.954<br>0.866	Dominant<br>Recessive<br>Additive
Weight, kg	70.5 (35.5 – 118.0)	74.4 (31.0 – 196.0)	73.1 (39.6 – 140.0)	0.499<br>0.048<br>0.097	Dominant<br>Recessive<br>Additive
Height, m	1.67 (1.40 – 1.93)	1.68 (1.28 – 1.91)	1.68 (1.42 – 1.89)	0.284<br>0.270<br>0.181	Dominant<br>Recessive<br>Additive
BMI, kg/m^2	25.2 (14.7 – 37.4)	26.0 (14.3 – 59.2)	25.6 (16.5 – 44.2)	0.891<br>0.121<br>0.299	Dominant<br>Recessive<br>Additive

Laboratory data					
				0.347	Dominant
CRP, mg/L	5.30 (0.20 – 83.30)	5.95 (0.10 – 195.00)	5.05 (0.30 – 120.60)	0.693	Recessive
				0.615	Additive
				0.199	Dominant
Total cholesterol, mg/dL	168.5 (92.0 – 336.0)	170.0 (72.0 – 626.0)	176.0 (76.3 – 363.0)	0.131	Recessive
				0.278	Additive
				0.014	Dominant
HDL-cholesterol, mg/dL	40.0 (7.0 – 93.0)	41.0 (14.0 – 103.0)	37.5 (6.0 – 118.0)	0.412	Recessive
				0.089	Additive
				0.026	Dominant
LDL-cholesterol, mg/dL	91.0 (26.8 – 232.0)	94.2 (27.0 – 512.0)	100.5 (25.0 – 350.0)	0.019	Recessive
				0.060	Additive
				0.391	Dominant
TG, mg/dL	147.0 (35.0 – 652.0)	144.5 (29.8 – 856.0)	155.0 (26.0 – 544.0)	0.553	Recessive
				0.987	Additive
				0.683	Dominant
Ca, mg/dL	8.82 (6.70 – 12.25)	8.83 (6.01 – 11.70)	8.83 (6.65 – 12.76)	0.741	Recessive
				0.707	Additive
				0.336	Dominant
Phosphate, mg/dL	5.30 (2.53 – 11.27)	4.98 (2.03 – 11.14)	5.21 (1.75 – 10.30)	0.400	Recessive
				0.883	Additive
				0.850	Dominant
Intact PTH, pg/mL	369.9 (5.5 – 2991.5)	391.2 (12.9 – 3757.0)	389.8 (8.9 – 2186.0)	0.492	Recessive
				0.629	Additive
				0.367	Dominant
Calcium phosphate product, mg^2/dL^2	45.5 (19.3 – 105.3)	43.8 (16.7 – 90.7)	45.6 (17.6 – 108.7)	0.456	Recessive
				0.881	Additive

[1] Chi-squared test for dichotomous variables or Mann-Whitney U test for quantitative variables

Table S6: *FOXO3* rs4946936 nucleotide variants and clinical and laboratory data of HD patients (n = 778)

Parameter	TT<br>N = 74	CT<br>N = 324	CC<br>N = 380	P value[1]	
Clinical data					
Male gender, n (%)	45 (61%)	188 (58%)	205 (54%)	0.196<br>0.881<br>0.830	Dominant<br>Recessive<br>Additive
Age at the beginning of RRT, years	59.7 (18.6 – 91.7)	61.1 (8.7 – 90.8)	61.7 (11.1 – 89.4)	0.316<br>0.332<br>0.181	Dominant<br>Recessive<br>Additive
RRT vintage, years	7.11 (0.40 – 25.56)	5.42 (0.09 – 31.09)	6.07 (0.04 – 32.88)	0.306<br>0.054<br>0.777	Dominant<br>Recessive<br>Additive
Renal transplantation, n (%)	13 (18%)	73 (23%)	62 (16%)	0.060<br>0.737<br>0.790	Dominant<br>Recessive<br>Additive
Diabetic nephropathy, n (%)	23 (31%)	100 (31%)	115 (30%)	0.846<br>0.923<br>0.889	Dominant<br>Recessive<br>Additive
Hypertensive nephropathy, n (%)	14 (19%)	72 (22%)	79 (21%)	0.780<br>0.613<br>0.713	Dominant<br>Recessive<br>Additive
Chronic glomerulonephritis, n (%)	10 (14%)	38 (12%)	54 (14%)	0.374<br>0.914<br>0.875	Dominant<br>Recessive<br>Additive
Chronic tubulointerstitial nephritis, n (%)	5 (7%)	30 (9%)	27 (7%)	0.385<br>0.686<br>0.915	Dominant<br>Recessive<br>Additive
Coronary artery disease, n (%)	29 (39%)	114 (35%)	150 (39%)	0.344<br>0.608<br>0.864	Dominant<br>Recessive<br>Additive
Myocardial infarction, n (%)	18 (24%)	61 (19%)	84 (22%)	0.440<br>0.454<br>0.676	Dominant<br>Recessive<br>Additive
Cerebral stroke, n (%)	14 (19%)	90 (28%)	100 (26%)	0.953<br>0.133<br>0.179	Dominant<br>Recessive<br>Additive
Dyslipidemia by K/DOQI, n (%)	36 (49%)	154 (48%)	217 (57%)	0.009<br>0.507<br>0.180	Dominant<br>Recessive<br>Additive
Hypolipemic therapy, n (%)	29 (39%)	135 (42%)	161 (42%)	0.742<br>0.636<br>0.612	Dominant<br>Recessive<br>Additive
Smoker, n (%)	10 (14%)	49 (15%)	72 (19%)	0.421<br>0.125<br>0.266	Dominant<br>Recessive<br>Additive
Weight, kg	70.4 (39.3 – 110.0)	73.2 (35.5 - 196)	74.0 (31.0 – 122.0)	0.629<br>0.174<br>0.458	Dominant<br>Recessive<br>Additive
Height, m	1.68 (1.28 – 1.86)	1.68 (1.40 – 1.90)	1.68 (1.36 – 1.93)	0.779<br>0.794<br>0.885	Dominant<br>Recessive<br>Additive
BMI, kg/m^2	24.3 (17.4 – 37.9)	26.0 (14.7 – 59.2)	25.7 (14.3 – 39.8)	0.550<br>0.104<br>0.276	Dominant<br>Recessive<br>Additive

Laboratory data					
				0.383	Dominant
CRP, mg/L	5.45 (0.60 – 142.00)	5.70 (0.10 – 181.00)	5.30 (0.20 – 119.60)	0.739	Recessive
				0.352	Additive
				0.029	Dominant
Total cholesterol, mg/dL	168.5 (84.9 – 336.0)	167.5 (76.3 – 626.0)	176.0 (72.0 – 363.0)	0.488	Recessive
				0.146	Additive
				0.791	Dominant
HDL-cholesterol, mg/dL	40.9 (17.3 – 118.0)	39.0 (7.0 – 103.0)	40.0 (6.0 – 90.2)	0.222	Recessive
				0.477	Additive
				0.036	Dominant
LDL-cholesterol, mg/dL	96.0 (36.1 – 215.0)	92.0 (25.0 – 512.0)	99.7 (27.0 – 369.0)	0.609	Recessive
				0.173	Additive
				0.973	Dominant
TG, mg/dL	147.5 (29.8 – 475.0)	150.5 (39.0 – 691.0)	146.9 (26.0 – 856.0)	0.404	Recessive
				0.956	Additive
				0.452	Dominant
Ca, mg/dL	8.71 (6.65 – 12.25)	8.81 (6.01 – 12.76)	8.87 (6.74 – 11.70)	0.634	Recessive
				0.743	Additive
				0.063	Dominant
Phosphate, mg/dL	5.03 (2.78 – 10.30)	4.98 (2.03 – 10.96)	5.30 (1.75 – 11.27)	0.691	Recessive
				0.193	Additive
				0.619	Dominant
Intact PTH, pg/mL	418.9 (7.3 – 3118.3)	390.7 (9.7 – 3757.0)	383.6 (5.5 – 3000.0)	0.697	Recessive
				0.636	Additive
				0.059	Dominant
Calcium phosphate product, mg^2/dL^2	43.5 (22.6 – 108.7)	43.8 (18.4 – 100.5)	45.7 (16.7 – 105.3)	0.774	Recessive
				0.248	Additive

[1] Chi-squared test for dichotomous variables or Mann-Whitney U test for quantitative variables

Table S7: Associations between the selected energy homeostasis nucleotide variants and all-cause mortality of HD patients evaluated by Kaplan-Meier analysis

**Tested variant**	**N**	**Major homozygotes vs. heterozygotes vs. minor homozygotes**[1]	**Dominant mode of inheritance**[2]	**Recessive mode of inheritance**[2]	**Additive mode of inheritance**[2]
FOXO3 rs2802292	784	TT vs. GT vs. GG<br>p = 0.9	GG + GT vs. TT<br>p = 0.9	GG vs. GT + TT<br>p = 0.7	GG vs. TT<br>p = 0.9
FOXO3 rs4946936	778	CC vs. CT vs. TT<br>p = 0.3	TT + CT vs. CC<br>p = 0.7	TT vs. CT + CC<br>p = 0.1	TT vs. CC<br>P = 0.1
IGFBP3 rs3110697	780	GG vs. AG vs. AA<br>p = 0.4	AA + AG vs. GG<br>p = 0.2	AA vs. AG + GG<br>p = 0.7	AA vs. GG<br>p = 0.3
IGFBP3 rs2854744	782	GG vs. GT vs. TT<br>p = 0.7	TT + GT vs. GG<br>p = 0.9	TT vs. GT + GG<br>p = 0.4	TT vs. GG<br>p = 0.6
FABP1 rs2241883	782	TT vs. CT vs. CC<br>p = 0.6	CC + CT vs. TT<br>p = 0.5	CC vs. CT + TT<br>p = 0.6	CC vs. TT<br>p = 0.9
FABP1 rs2919872	775	CC vs. CT vs. TT<br>p = 0.5	TT + CT vs. CC<br>p = 0.7	TT vs. CT + CC<br>p = 0.3	TT vs. CC<br>p = 0.6
PCSK9 rs562556	780	AA vs. AG vs. GG<br>p = 0.4	GG + AG vs. AA<br>p = 1.0	GG vs. AG + AA<br>p = 0.2	GG vs. AA<br>p = 0.2
PCSK9 rs11206510	781	TT vs. CT vs. CC<br>p = 0.1	CC + CT vs. TT<br>p = 0.9	CC vs. CT + TT<br>p = 0.4	CC vs. TT<br>p = 0.4
ANGPTL6 rs8112063	780	TT vs. CT vs. CC<br>p = 0.1	CC + CT vs. TT<br>p = 0.05	CC vs. CT + TT<br>p = 0.6	CC vs. TT<br>p = 0.2
DOCK6 rs737337	780	TT vs. CT vs. CC<br>p = 0.03	CC + CT vs. TT<br>p = 0.07	CC vs. CT + TT<br>p = 0.02	CC vs. TT<br>p = 0.02
DOCK6 rs17699089	783	AA vs. AG vs. GG<br>p = 0.2	GG + AG vs. AA<br>p = 0.06	GG vs. AG + AA<br>p = 0.6	GG vs. AA<br>p = 0.5

[1] Multiple-sample test p
[2] Log-rank test p

Table S8 Associations between the selected energy homeostasis nucleotide variants and cardiovascular mortality of HD patients evaluated by Kaplan-Meier analysis

Tested variant	N	Major homozytes vs. heterozygotes vs. minor homozygotes[1]	Dominant mode of inheritance[2]	Recessive mode of inheritance[2]	Additive mode of inheritance[2]
FOXO3 rs2802292	784	TT vs. GT vs. GG<br>p = 0.7	GG + GT vs. TT<br>p = 0.4	GG vs. GT + TT<br>p = 0.7	GG vs. TT<br>p = 0.5
FOXO3 rs4946936	778	CC vs. CT vs. TT<br>p = 0.1	TT + CT vs. CC<br>p = 0.9	TT vs. CT + CC<br>p = 0.04	TT vs. CC<br>p = 0.06
IGFBP3 rs3110697	780	GG vs. AG vs. AA<br>p = 0.4	AA + AG vs. GG<br>p = 0.2	AA vs. AG + GG<br>p = 0.4	AA vs. GG<br>p = 0.2
IGFBP3 rs2854744	782	GG vs. GT vs. TT<br>p = 0.4	TT + GT vs. GG<br>p = 0.8	TT vs. GT + GG<br>p = 0.3	TT vs. GG<br>p = 0.5
FABP1 rs2241883	782	TT vs. CT vs. CC<br>p = 0.8	CC + CT vs. TT<br>p = 0.5	CC vs. CT + TT<br>p = 0.9	CC vs. TT<br>p = 0.7
FABP1 rs2919872	775	CC vs. CT vs. TT<br>p = 0.8	TT + CT vs. CC<br>p = 0.6	TT vs. CT + CC<br>p = 0.8	TT vs. CC<br>p = 0.6
PCSK9 rs562556	780	AA vs. AG vs. GG<br>p = 0.6	GG + AG vs. AA<br>p = 0.3	GG vs. AG + AA<br>p = 0.6	GG vs. AA<br>p =0.6
PCSK9 rs11206510	781	TT vs. CT vs. CC<br>p = 0.9	CC + CT vs. TT<br>p = 1.0	CC vs. CT + TT<br>p = 0.6	CC vs. TT<br>p = 0.6
ANGPTL6 rs8112063	780	TT vs. CT vs. CC<br>p = 0.04	CC + CT vs. TT<br>p = 0.01	CC vs. CT + TT<br>p = 0.5	CC vs. TT<br>p = 0.07
DOCK6 rs737337	780	TT vs. CT vs. CC<br>p = 0.2	CC + CT vs. TT<br>p = 0.2	CC vs. CT + TT<br>p = 0.09	CC vs. TT<br>p = 0.09
DOCK6 rs17699089	783	AA vs. AG vs. GG<br>p = 0.4	GG + AG vs. AA<br>p = 0.5	GG vs. AG + AA<br>p = 0.4	GG vs. AA<br>p = 0.4

[1] Multiple-sample test p
[2] Log-rank test p

Table S9: Associations between the selected energy homeostasis nucleotide variants and cardiac mortality of HD patients evaluated by Kaplan-Meier analysis

Tested variant	N	Major homozytes vs. heterozygotes vs. minor homozygotes[1]	Dominant mode of inheritance[2]	Recessive mode of inheritance[2]	Additive mode of inheritance[2]
FOXO3 rs2802292	784	TT vs. GT vs. GG<br>p = 0.5	GG + GT vs. TT<br>p = 0.5	GG vs. GT + TT<br>p = 0.3	GG vs. TT<br>p = 0.2
FOXO3 rs4946936	778	CC vs. CT vs. TT<br>p = 0.06	TT + CT vs. CC<br>p = 0.7	TT vs. CT + CC<br>p = 0.04	TT vs. CC<br>p = 0.07
IGFBP3 rs3110697	780	GG vs. AG vs. AA<br>p = 0.3	AA + AG vs. GG<br>p = 0.2	AA vs. AG + GG<br>p = 0.3	AA vs. GG<br>p = 0.2
IGFBP3 rs2854744	782	GG vs. GT vs. TT<br>p = 0.7	TT + GT vs. GG<br>p = 0.8	TT vs. GT + GG<br>p = 0.4	TT vs. GG<br>p = 0.5
FABP1 rs2241883	782	TT vs. CT vs. CC<br>p = 0.7	CC + CT vs. TT<br>p = 0.8	CC vs. CT + TT<br>p = 0.5	CC vs. TT<br>p = 0.7
FABP1 rs2919872	775	CC vs. CT vs. TT<br>p = 0.9	TT + CT vs. CC<br>p = 0.8	TT vs. CT + CC<br>p = 0.6	TT vs. CC<br>p = 0.6
PCSK9 rs562556	780	AA vs. AG vs. GG<br>p = 0.5	GG + AG vs. AA<br>p = 0.2	GG vs. AG + AA<br>p = 0.9	GG vs. AA<br>p =0.9
PCSK9 rs11206510	781	TT vs. CT vs. CC<br>p = 0.6	CC + CT vs. TT<br>p = 0.4	CC vs. CT + TT<br>p = 0.9	CC vs. TT<br>p = 0.8
ANGPTL6 rs8112063	780	TT vs. CT vs. CC<br>p = 0.01	CC + CT vs. TT<br>p = 0.003	CC vs. CT + TT<br>p = 0.2	CC vs. TT<br>p = 0.01
DOCK6 rs737337	780	TT vs. CT vs. CC<br>p = 0.04	CC + CT vs. TT<br>p = 0.05	CC vs. CT + TT<br>p = 0.05	CC vs. TT<br>p = 0.03
DOCK6 rs17699089	783	AA vs. AG vs. GG<br>p = 0.1	GG + AG vs. AA<br>p = 0.07	GG vs. AG + AA<br>p = 0.8	GG vs. AA<br>p = 1.0

[1] Multiple-sample test p
[2] Log-rank test p

Table S10: Associations between the selected energy homeostasis nucleotide variants and all-cause mortality of patients initiating HD therapy evaluated by Kaplan-Meier analysis

**Tested variant**	**N**	**Major homozygotes vs. heterozygotes vs. minor homozygotes**[1]	**Dominant mode of inheritance**[2]	**Recessive mode of inheritance**[2]	**Additive mode of inheritance**[2]
FOXO3 rs2802292	82	TT vs. GT vs. GG<br>p = 0.5	GG + GT vs. TT<br>p = 0.4	GG vs. GT + TT<br>p = 0.4	GG vs. TT<br>p = 0.3
FOXO3 rs4946936	80	CC vs. CT vs. TT<br>p = 10.9	TT + CT vs. CC<br>p = 0.7	TT vs. CT + CC<br>p = 0.7	TT vs. CC<br>P = 0.7
IGFBP3 rs3110697	82	GG vs. AG vs. AA<br>p = 0.5	AA + AG vs. GG<br>p = 0.4	AA vs. AG + GG<br>p = 0.8	AA vs. GG<br>p = 0.9
IGFBP3 rs2854744	77	GG vs. GT vs. TT<br>p = 0.5	TT + GT vs. GG<br>p = 0.7	TT vs. GT + GG<br>p = 0.2	TT vs. GG<br>p = 0.3
FABP1 rs2241883	83	TT vs. CT vs. CC<br>p = 0.5	CC + CT vs. TT<br>p = 0.5	CC vs. CT + TT<br>p = 0.5	CC vs. TT<br>p = 1.0
FABP1 rs2919872	80	CC vs. CT vs. TT<br>p = 0.06	TT + CT vs. CC<br>p = 0.04 (p_{corr} = 0.05)	TT vs. CT + CC<br>p = 0.1	TT vs. CC<br>p = 0.06
PCSK9 rs562556	82	AA vs. AG vs. GG<br>p = 0.09	GG + AG vs. AA<br>p = 0.05	GG vs. AG + AA<br>p = 0.4	GG vs. AA<br>p = 0.6
PCSK9 rs11206510	82	TT vs. CT vs. CC<br>p = 0.1	CC + CT vs. TT<br>p = 0.09	CC vs. CT + TT<br>p = 0.4	CC vs. TT<br>p = 0.5
ANGPTL6 rs8112063	82	TT vs. CT vs. CC<br>p = 0.8	CC + CT vs. TT<br>p = 0.9	CC vs. CT + TT<br>p = 0.5	CC vs. TT<br>p = 0.7
DOCK6 rs737337	81	TT vs. CT vs. CC<br>p = 0.01 (p_{corr} = 0.1)	CC + CT vs. TT<br>p = 0.2	CC vs. CT + TT<br>p = 0.006 (p_{corr} = 0.08)	CC vs. TT<br>p = 0.006 (p_{corr} = 0.09)
DOCK6 rs17699089	80	AA vs. AG vs. GG<br>p = 0.007 (p_{corr} = 0.02)	GG + AG vs. AA<br>p = 0.09	GG vs. AG + AA<br>p = 0.002 (p_{corr} = 0.04)	GG vs. AA<br>p = 0.001 (p_{corr} = 0.03)

[1] Multiple-sample test p
[2] Log-rank test p

Table S11: Associations between the selected energy homeostasis nucleotide variants and cardiovascular mortality of patients initiating HD therapy evaluated by Kaplan-Meier analysis

Tested variant	N	Major homozygotes vs. heterozygotes vs. minor homozygotes[1]	Dominant mode of inheritance[2]	Recessive mode of inheritance[2]	Additive mode of inheritance[2]
FOXO3 rs2802292	82	TT vs. GT vs. GG<br>$p = 0.4$	GG + GT vs. TT<br>$p = 0.4$	GG vs. GT + TT<br>$p = 0.2$	GG vs. TT<br>$p = 0.2$
FOXO3 rs4946936	80	CC vs. CT vs. TT<br>$p = 0.7$	TT + CT vs. CC<br>$p = 0.4$	TT vs. CT + CC<br>$p = 0.8$	TT vs. CC<br>$P = 0.7$
IGFBP3 rs3110697	82	GG vs. AG vs. AA<br>$p = 0.05$	AA + AG vs. GG<br>$p = 0.01$ ($p_{corr} =1.0$)	AA vs. AG + GG<br>$p = 0.6$	AA vs. GG<br>$p = 0.05$
IGFBP3 rs2854744	77	GG vs. GT vs. TT<br>$p = 0.1$	TT + GT vs. GG<br>$p = 0.2$	TT vs. GT + GG<br>$p = 0.05$	TT vs. GG<br>$p = 0.04$ ($p_{corr} =1.0$)
FABP1 rs2241883	83	TT vs. CT vs. CC<br>$p = 0.3$	CC + CT vs. TT<br>$p = 0.9$	CC vs. CT + TT<br>$p = 0.2$	CC vs. TT<br>$p = 0.5$
FABP1 rs2919872	80	CC vs. CT vs. TT<br>$p = 0.4$	TT + CT vs. CC<br>$p = 0.2$	TT vs. CT + CC<br>$p = 0.5$	TT vs. CC<br>$p = 0.4$
PCSK9 rs562556	82	AA vs. AG vs. GG<br>$p = 0.01$ ($p_{corr} = 0.06$)	GG + AG vs. AA<br>$p = 0.07$	GG vs. AG + AA<br>$p = 0.03$ ($p_{corr} = 0.08$)	GG vs. AA<br>$p = 0.1$
PCSK9 rs11206510	82	TT vs. CT vs. CC<br>$p = 0.03$ ($p_{corr} = 0.09$)	CC + CT vs. TT<br>$p = 0.07$	CC vs. CT + TT<br>$p = 0.1$	CC vs. TT<br>$p = 0.2$
ANGPTL6 rs8112063	82	TT vs. CT vs. CC<br>$p = 0.9$	CC + CT vs. TT<br>$p = 0.8$	CC vs. CT + TT<br>$p = 0.7$	CC vs. TT<br>$p = 0.7$
DOCK6 rs737337	81	TT vs. CT vs. CC<br>$p < 0.001$ ($p_{corr} = 0.02$)	CC + CT vs. TT<br>$p = 0.4$	CC vs. CT + TT<br>$p < 0.001$ ($p_{corr} = 0.01$)	CC vs. TT<br>$p < 0.001$ ($p_{corr} = 0.02$)
DOCK6 rs17699089	80	AA vs. AG vs. GG<br>$p < 0.001$ ($p_{corr} = 0.01$)	GG + AG vs. AA<br>$p = 0.9$	GG vs. AG + AA<br>$p < 0.001$ ($p_{corr} = 0.03$)	GG vs. AA<br>$p < 0.001$ ($p_{corr} = 0.04$)

[1] Multiple-sample test p

[2] Log-rank test p

Table S12: Associations between the selected energy homeostasis nucleotide variants and cardiac mortality of patients initiating HD therapy evaluated by Kaplan-Meier analysis

Tested variant	N	Major homozygotes vs. heterozygotes vs. minor homozygotes[1]	Dominant mode of inheritance[2]	Recessive mode of inheritance[2]	Additive mode of inheritance[2]
FOXO3 rs2802292	82	TT vs. GT vs. GG<br>p = 0.7	GG + GT vs. TT<br>p = 0.4	GG vs. GT + TT<br>p = 0.8	GG vs. TT<br>p = 0.6
FOXO3 rs4946936	80	CC vs. CT vs. TT<br>p = 0.7	TT + CT vs. CC<br>p = 0.7	TT vs. CT + CC<br>p = 0.4	TT vs. CC<br>P = 0.5
IGFBP3 rs3110697	82	GG vs. AG vs. AA<br>p = 0.02 (p_{corr}=0.9)	AA + AG vs. GG<br>p = 0.009 (p_{corr}=1.0)	AA vs. AG + GG<br>p = 0.9	AA vs. GG<br>p = 0.05
IGFBP3 rs2854744	77	GG vs. GT vs. TT<br>p = 0.2	TT + GT vs. GG<br>p = 0.2	TT vs. GT + GG<br>p = 0.08	TT vs. GG<br>p = 0.06
FABP1 rs2241883	83	TT vs. CT vs. CC<br>p = 0.7	CC + CT vs. TT<br>p = 0.6	CC vs. CT + TT<br>p = 0.4	CC vs. TT<br>p = 0.4
FABP1 rs2919872	80	CC vs. CT vs. TT<br>p = 0.4	TT + CT vs. CC<br>p = 0.2	TT vs. CT + CC<br>p = 0.9	TT vs. CC<br>p = 0.8
PCSK9 rs562556	82	AA vs. AG vs. GG<br>p = 0.01 (p_{corr} = 0.06)	GG + AG vs. AA<br>p = 0.2	GG vs. AG + AA<br>p = 0.01 (p_{corr} = 0.08)	GG vs. AA<br>p = 0.05
PCSK9 rs11206510	82	TT vs. CT vs. CC<br>p = 0.05	CC + CT vs. TT<br>p = 0.2	CC vs. CT + TT<br>p = 0.07	CC vs. TT<br>p = 0.1
ANGPTL6 rs8112063	82	TT vs. CT vs. CC<br>p = 0.8	CC + CT vs. TT<br>p = 0.5	CC vs. CT + TT<br>p = 0.6	CC vs. TT<br>p = 0.5
DOCK6 rs737337	81	TT vs. CT vs. CC<br>p < 0.001 (p_{corr} = 0.02)	CC + CT vs. TT<br>p = 0.2	CC vs. CT + TT<br>p < 0.001 (p_{corr} = 0.01)	CC vs. TT<br>p < 0.001 (p_{corr} = 0.01)
DOCK6 rs17699089	80	AA vs. AG vs. GG<br>p < 0.001 (p_{corr} = 0.005)	GG + AG vs. AA<br>p = 0.5	GG vs. AG + AA<br>p < 0.001 (p_{corr} = 0.02)	GG vs. AA<br>p < 0.001 (p_{corr} = 0.03)

[1] Multiple-sample test p
[2] Log-rank test p

Table S13: Frequency distributions of the analyzed nucleotide variants in hemodialysis patients in which protein analyses were performed and in those without protein analysis

Gene symbol	rs number	Patients with measured protein plasma concentrations			Patients without measured protein plasma concentrations			P value[1]	
		Major homozygotes	Heterozygotes	Minor homozygotes	Major homozygotes	Heterozygotes	Minor homozygotes		
								0.633	Dominant
FOXO3	rs2802292	31 (36.9%)	39 (46.4%)	14 (16.7%)	240 (34.2%)	357 (51.0%)	103 (14.7%)	0.635	Recessive
								0.510	Additive
								0.359	Dominant
FOXO3	rs4946936	45 (53.6%)	28 (33.3%)	11 (13.1%)	335 (48.3%)	296 (42.7%)	63 (9.1%)	0.236	Recessive
								0.469	Additive
								0.998	Dominant
IGFBP3	rs3110697	29 (34.1%)	40 (47.1%)	16 (18.8%)	237 (34.1%)	337 (48.5%)	121 (17.4%)	0.747	Recessive
								0.815	Additive
								0.504	Dominant
IGFBP3	rs2854744	33 (38.8%)	40 (47.0%)	12 (14.1%)	245 (35.2%)	336 (48.2%)	116 (16.6%)	0.553	Recessive
								0.457	Additive
								0.768	Dominant
FABP1	rs2241883	28 (33.3%)	41 (48.8%)	15 (17.9%)	244 (35.0%)	336 (48.1%)	118 (16.9%)	0.826	Recessive
								0.763	Additive
								0.873	Dominant
FABP1	rs2919872	26 (30.1%)	44 (52.4%)	14 (16.7%)	208 (30.1%)	343 (49.6%)	140 (20.3%)	0.436	Recessive
								0.522	Additive
								0.448	Dominant
PCSK9	rs562556	54 (63.5%)	28 (32.9%)	3 (3.5%)	470 (67.6%)	212 (30.5%)	13 (1.9%)	0.404[2]	Recessive
								0.233[2]	Additive
								0.274	Dominant
PCSK9	rs11206510	64 (75.2%)	16 (18.8%)	5 (5.9%)	484 (69.5%)	194 (27.9%)	18 (2.6%)	0.094[a]	Recessive
								0.181[2]	Additive
ANGPTL6	rs8112063	34 (40%)	39 (45.9%)	12 (14.1%)	204 (29.4%)	331 (47.6%)	160 (23.0%)	0.062	Dominant
								0.021	Recessive

								0.018	Additive
DOCK6	rs737337	69 (83.1%)	12 (14.5%)	2 (2.4%)	556 (79.8%)	129 (18.5%)	12 (1.7%)	0.468	Dominant
								0.653[2]	Recessive
								0.662[2]	Additive
DOCK6	rs176990893	61 (74.4%)	15 (18.3%)	6 (7.3%)	492 (70.2%)	184 (26.2%)	25 (3.6%)	0.429	Dominant
								0.125[2]	Recessive
								0.152[2]	Additive

[1] Chi-squared test
[2] Fisher's exact test

Table S14: Correlations between the plasma concentrations of the studied proteins and survival probability of HD patients in a 3.5-year prospective analysis from June 2016 (n = 86)

**Tested protein**	**All-cause mortality**[1]	**Cardiovascular mortality**[1]	**Cardiac mortality**[1]
FOXO3	p = 0.1	p = 0.1	p = 1.0
IGFBP-3	p = 0.2	p = 0.6	p = 1.0
L-FABP	p = 0.2	p = 0.3	p = 0.3
PCSK9	p = 0.9	p = 0.2	p = 1.0
ANGPTL6	p = 0.8	p = 0.8	p = 0.09
ANGPTL8	p = 0.7	p = 0.9	p = 0.7

[1] Wald test statistic using a proportional hazards model of Cox

Table S15: Comparison of the analyzed nucleotide variants and the serum concentrations of their protein products in HD patients (n = 86)

Protein	Gene symbol	rs number	Protein concentrations				
			Major homozygotes	Heterozygotes	Minor homozygotes	P value[1]	
FOXO3 [ng/ml]	*FOXO3*	rs2802292	65.1 (19.8 – 181.3)	66.3 (14.4 – 284.4)	62.3 (19.0 – 139.6)	0.933	Dominant
						0.577	Recessive
						0.879	Additive
FOXO3 [ng/ml]	*FOXO3*	rs4946936	73.8 (19.8 – 234.2)	55.3 (14.4 – 284.4)	68.4 (19.0 – 165.1)	0.260	Dominant
						0.720	Recessive
						0.607	Additive
IGFBP-3 [ug/ml]	*IGFBP3*	rs3110697	2.69 (0.61 – 4.43)	2.02 (1.17 – 6.27)	2.36 (1.18 – 5.97)	0.270	Dominant
						0.831	Recessive
						0.853	Additive
IGFBP-3 [ug/ml]	*IGFBP3*	rs2854744	1.99 (1.17 – 6.27)	2.29 (0.61 – 4.25)	2.75 (2.03 – 4.43)	0.243	Dominant
						0.061	Recessive
						0.336	Additive
L-FABP [ng/ml]	*FABP1*	rs2241883	36.9 (4.0 – 114.3)	35.2 (5.2 – 107.4)	57.9 (13.5 – 91.6)	0.331	Dominant
						0.023	Recessive
						0.085	Additive
L-FABP [ng/ml]	*FABP1*	rs2919872	40.3 (4.0 – 114.3)	44.9 (5.2 – 91.6)	22.4 (9.9 – 88.8)	0.400	Dominant
						0.037	Recessive
						0.074	Additive
PCSK9 [ng/ml]	*PCSK9*	rs562556	388.1 (182.7 – 747.0)	389.2 (178.5 – 621.1)	443.6 (191.2 – 522.3)	0.776	Dominant
						0.922	Recessive
						0.847	Additive
PCSK9 [ng/ml]	*PCSK9*	rs11206510	408.1 (178.5 – 631.7)	369.2 (182.7 – 570.6)	372.4 (335.2 – 747.0)	0.886	Dominant
						0.367	Recessive
						0.310	Additive
ANGPTL6 [ng/ml]	*ANGPTL6*	rs8112063	150.9 (39.9 – 368.7)	168.9 (60.9 – 497.2)	134.4 (74.2 – 345.6)	0.235	Dominant
						0.729	Recessive
						0.354	Additive

						0.312	Dominant
ANGPTL8 [pg/ml]	*DOCK6*	rs737337	994.6 (69.2 – 2604.4)	853.6 (259.4 – 1771.2)	1414.5 (1359.2 – 1469.9)	0.119	Recessive
						0.598	Additive
						0.770	Dominant
ANGPTL8 [pg/ml]	*DOCK6*	rs176990893	947.6 (69.2 – 2604.4)	969.6 (259.4 – 1771.2)	1113.9 (566.9 – 1469.9)	0.769	Recessive
						0.682	Additive

[1] Mann-Whitney U test

Table S16: Epistatic interactions between the analyzed genes with respect to dyslipidemia by K/DOQI in HD patients

No. of risk genes	Models	Testing balanced accuracy	CVC	OR-MDR	95%CI	p-value[1]
2	*FOXO3 rs2802292_ FABP1 rs2241883*	0.59	6/10	0.472	(0.238 - 0.934)	0.021
5	*FOXO3 rs2802292_ IGFBP3 rs3110697_ FABP1 rs2241883_ FABP1 rs2919872_ ANGPTL6 rs8112063*	0.52	8/10	0.302	(0.032 - 2.886)	0.275

Abbreviations: *CVC* Cross-validation consistency; *OR-MDR* odds ratio-multifactor-dimensionality reduction; *95% CI* confidence interval 95%.

Table S17: Epistatic interactions between the analyzed genes with respect to cardiac mortality in HD patients

No. of risk genes	Models	Testing balanced accuracy	CVC	OR-MDR	95%CI	p-value[1]
2	*FOXO3 rs2802292_ DOCK6 rs737337*	0.66	4/10	0.577	(0.168 - 1.988)	0.279
3	*FOXO3 rs2802292_ DOCK6 rs737337_ DOCK6 rs17699089*	0.63	2/10	0.337	(0.042 - 2.676)	0.252
4	*PCSK9 rs562556 _ PCSK9 rs11206510 _ ANGPTL6 rs8112063 _ DOCK6 rs737337*	0.64	4/10	0.539	(0.063 - 4.585)	0.486
5	*IGFBP3 rs2854744_* PCSK9 rs562556 _ PCSK9 rs11206510 _ *ANGPTL6 rs8112063_ DOCK6 rs737337*	0.56	3/10	0.270	(0.035 - 2.092)	0.151

Table S18: Epistatic interactions between the analyzed genes with respect to CAD in HD patients

No. of risk genes	Models	Testing balanced accuracy	CVC	OR-MDR	95%CI	p-value[1]
2	*IGFBP3 rs2854744_ DOCK6 rs737337*	0.58	4/10	0.322	(0.038 - 2.744)	0.259
3	*FOXO3 rs2802292_ FOXO3 rs4946936_ FABP1 rs2919872*	0.56	3/10	0.537	(0.197 - 1.462)	0.157
4	*FOXO3 rs4946936_* IGFBP3 rs2854744 *_FABP1 rs2919872_ DOCK6 rs737337*	0.59	9/10	0.322	(0.071 - 1.460)	0.101

Table S19: Epistatic interactions between the analyzed genes with respect to diabetes in HD patients

No. of risk genes	Models	Testing balanced accuracy	CVC	OR-MDR	95%CI	p-value[1]
2	*DOCK6 rs737337_ DOCK6 rs17699089*	0.64	3/10	2.241	(0.318 - 15.812)	0.914
3	*IGFBP3 rs3110697_ IGFBP3 rs2854744_ DOCK6 rs737337*	0.64	3/10	0.659	(0.246" "1.765)	0.280
4	*IGFBP3 rs3110697_* FABP1 rs2919872_ *ANGPTL6 rs8112063_ DOCK6 rs737337*	0.60	3/10	0.320	(0.073 - 1.397)	0.085

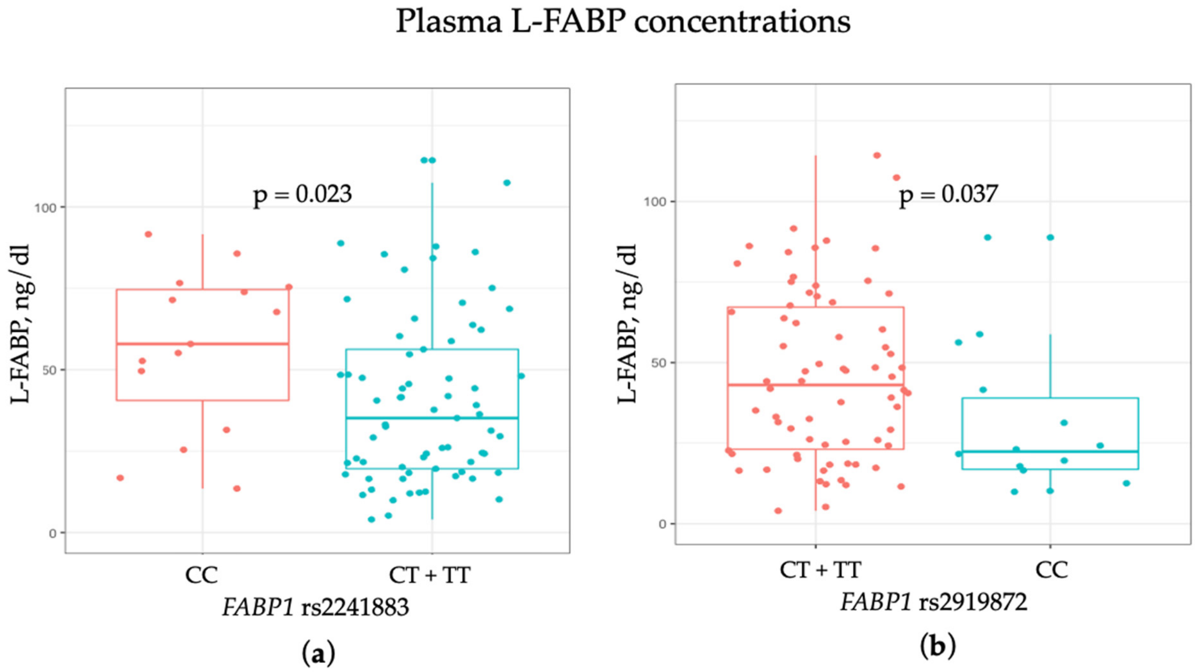

Figure S1: L-FABP plasma concentrations according to the analyzed nucleotide variants: (**a**) *FABP1* rs2241883 in the recessive mode of inheritance; (**b**) *FABP1* rs2919872 in the dominant mode of inheritance
